# Characterization and phylogenetic analysis of the complete plastome of *Ipomoea aquatica* (Convolvulaceae), an edible vegetable

**DOI:** 10.1080/23802359.2021.1891985

**Published:** 2021-03-18

**Authors:** Qing-Jun Wang, Rong Wang, Luo-yan Zhang, Xue-Jie Zhang

**Affiliations:** Key Lab of Plant Stress Research, College of Life Sciences, Shandong Normal University, Ji’nan, Shandong, China

**Keywords:** *Ipomoea aquatica*, plastome, phylogenomics

## Abstract

*Ipomoea aquatica*, commonly known as water spinach, is an edible annual vegetable in the genus *Ipomoea*,. In this study, the complete plastome of *Ipomoea aquatica* was determined using the Illumina sequencing platform. The plastome size was 162,663 bp. It consists of four regions, including a large single-copy region (88,166 bp), a small single-copy region (12,069 bp), and a pair of inverted repeat regions (31,214 bp). This plastome encodes 114 unique genes, including 80 protein-coding genes (PCGs), 30 transfer RNA genes (tRNAs), and 4 ribosomal RNA genes (rRNAs). The GC content was 39.1%. Phylogenomic analysis based on 19 complete plastomes revealed that *I. aquatica* was closely related to *I. diamantinensis*.

*Ipomoea aquatica* Forsskal, commonly known as water spinach, is an annual herb and can be used as a kind of vegetable for human consumption (Gothberg et al. 2002; Prasad et al. 2005, 2008). Water spinach originated from tropical and rainy areas of China. It is widely planted in China. It grows naturally in Asia and Southwest Pacific islands in summer and autumn (Edie and Bess 1969). Water spinach is rich in nutrients, including essential amino acids, vitamin A/C and iron (Umar et al. 2007; Marcussen et al. 2008). In addition, it is commonly used to study the heat tolerance of plants (Wang et al. 2019; Guo et al. 2020a). The latest transcriptome studies have shown that the response of metabolic pathways such as carbohydrate and phenylpropanoid biosynthesis to heat stress is a potential key factor in heat tolerance (Guo et al. 2020a). Heavy metals may pose a threat to human health through the food chain (Gu et al. 2003), so the study on physiological and biochemical reactions of water spinach under heavy metal conditions is also a hotspot (Xin et al. 2010; Zhang et al. 2011; Ton et al. 2015). With the development of technology, the application of sequencing technology to study plants is more and more common. Many characteristics of chloroplast genome size have been reported(Qu 2019; Qu et al. 2019b; Zhang et al. 2019a, 2019b; Guo et al. 2020b). But, there is no information about the size and structure of chloroplast genome in I. aquatic. There are few studies on the phylogeny of *I. aquatica* by using chloroplast genome data. In this study, we reported the plastome of *I. aquatica*, which would provide fundamental genetic resource for studying this important species.

The water spinach cultivars ‘Zhuye’ was sequenced in the study. Seeds of water spinach acquired from Zhihui Seed Industry Co. LTD (Henan, China) were cultivated in greenhouse. Voucher specimen (200910) was deposited at College of Life Sciences, Shandong Normal University. Fresh leaves of *I. aquatica* were used to extract the total genomic DNA by modified CTAB-based method. Total DNA were sent to Novogene (Beijing, China) for genomic library construction and Illumina sequencing. The read length was 150 bp, insert size 350 bp and total read number 9,494,413. Use the kit to build the Library (NEB Next Ultra DNA Library Prep Kit).The plastome was assembled with OGA (Organelle Genome Assembler) as described in (Qu et al. 2019a) and annotated with PGA (Plastid Genome Annotator) (Qu et al. 2019c). Geneious v8.0.2 (https://www.geneious.com) was used for manual check (Kearse et al. 2012). In order to determine the phylogenetic placement of *I. aquatica*, a maximum likelihood (ML) tree was reconstructed using RAxML v8.2.10 (Stamatakis 2014), including tree robustness assessment using 1000 rapid bootstrap replicates with the GTRGAMMA substitution model, based on the alignment of 78 protein-coding genes using MAFFT v7.471 (Katoh and Standley 2013).

The complete plastome of *I. aquatica* (GenBank accession number: MW250301) was 162,663 bp. It consists of four regions, including a large single-copy region (88,166 bp), a small single-copy region (12,069 bp), and a pair of inverted repeat regions (31,214 bp). It encodes 114 unique genes, including 80 protein-coding genes (PCGs), 30 transfer RNA genes (tRNAs), and 4 ribosomal RNA genes (rRNAs). The GC content was 39.1%. The chloroplast genome composition of *I. aquatica* is basically consistent with that of other species of *Ipomoea*, indicating that the chloroplast genome structure is relatively conservative. Phylogenomic analysis of 19 plastomes revealed that *I. aquatica* was closely related to *I. diamantinensis* (Figure 1).

**Figure 1.**
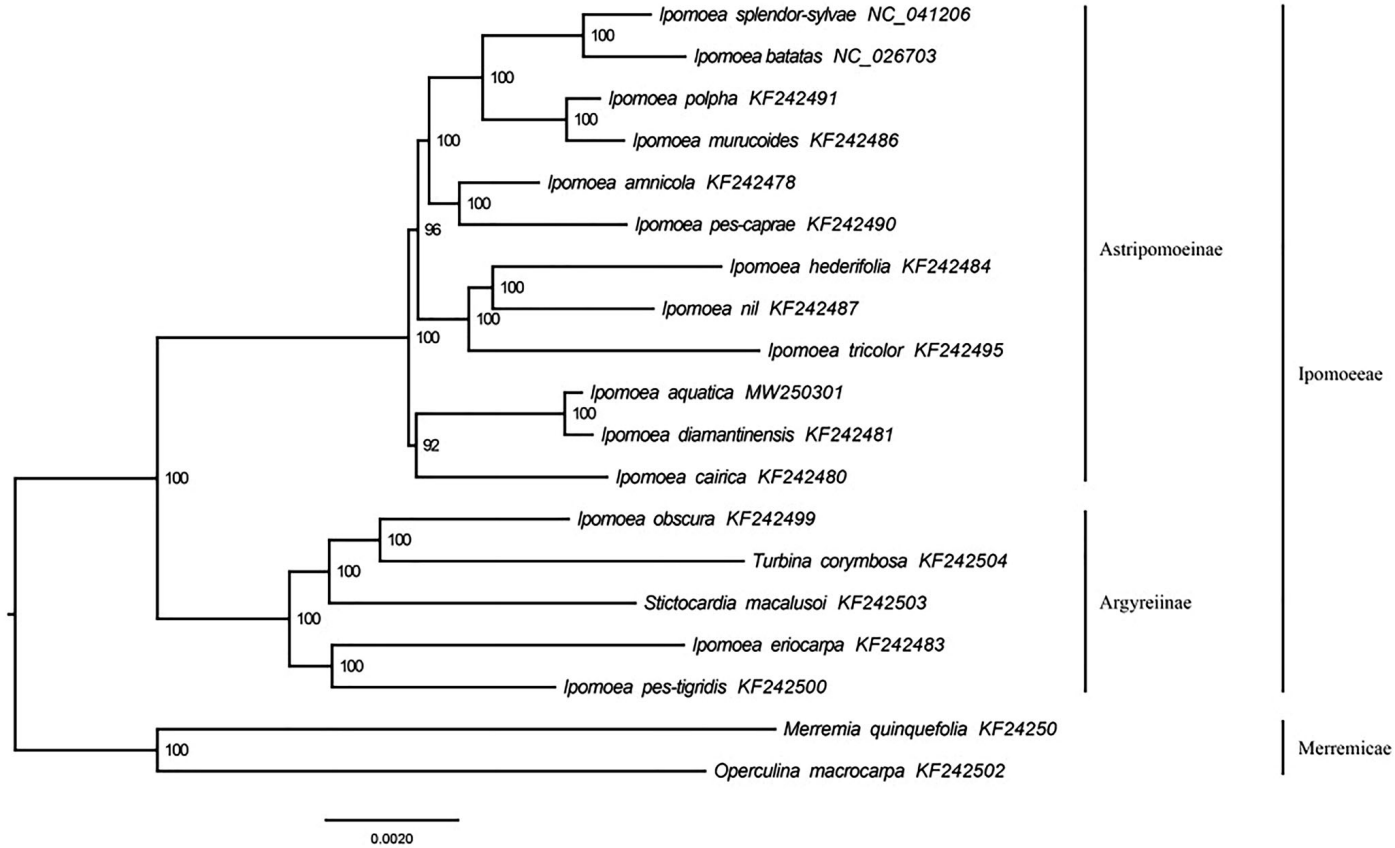
A maximum likelihood (ML) tree inferred from 19 complete plastomes is shown. The numbers next to nodes are bootstrap support values.

## Data Availability

The data that support the findings of this study is openly available in GenBank of NCBI at https://www.ncbi.nlm.nih.gov/nuccore/MW250301, reference number MW250301.SRA：https://www.ncbi.nlm.nih.gov/sra/PRJNA678848.
